# Ubigo-X: Protein ubiquitination site prediction using ensemble learning with image-based feature representation and weighted voting

**DOI:** 10.1016/j.csbj.2025.07.025

**Published:** 2025-07-14

**Authors:** Disline Manli Tantoh, Jen-Chieh Yu, Ching-Hsuan Chien, Wei-Yi Yeh, Yen-Wei Chu

**Affiliations:** a Doctoral Program in Medical Biotechnology, National Chung Hsing University, Taichung City, Taiwan; b Graduate Institute of Genomics and Bioinformatics, National Chung Hsing University, Taichung City, Taiwan; c Smart Sustainable New Agriculture Research Center (SMARTer), Taichung City, Taiwan; d Institute of Molecular Biology, National Chung Hsing University, Taichung City, Taiwan

**Keywords:** Protein ubiquitination, Machine learning, Deep learning, Ensemble learning, Weighted voting, Image-based feature representation

## Abstract

Accurate ubiquitination identification is crucial in biological function analysis. We developed Ubigo-X, a novel protein ubiquitination prediction tool. Our training data, sourced from the Protein Lysine Modification Database (PLMD 3.0), comprised 53,338 ubiquitination and 71,399 non-ubiquitination sites, retained after CD-HIT and CD-HIT-2d sequence filtering. Three sub-models: Single-Type sequence-based features (Single-Type SBF), k-mer sequence-based features (Co-Type SBF), and structure-based and function-based features (S-FBF), were developed. Single-Type SBF used amino acid composition (AAC), amino acid index (AAindex), and one-hot encoding; Co-Type SBF used Single-Type SBF via k-mer encoding; and S-FBF used secondary structure, relative solvent accessibility (RSA)/absolute solvent-accessible area (ASA), and signal peptide cleavage sites. S-FBF was trained using XGBoost, while Single-Type SBF and Co-Type SBF were transformed into image-based features and trained using Resnet34. Ubigo-X was developed by combining the three models via a weighted voting strategy. Independent testing using PhosphoSitePlus data (65,421 ubiquitination and 61,222 non-ubiquitination sites) retained after filtering yielded 0.85, 0.79, and 0.58 for area under the curve (AUC), accuracy (ACC), and Matthews correlation coefficient (MCC), respectively. Further testing on imbalanced PhosphoSitePlus data (1:8 positive-to-negative sample ratio) yielded 0.94 AUC, 0.85 ACC, and 0.55 MCC. Using the GPS-Uber data, the AUC, ACC, and MCC were 0.81, 0.59, and 0.27, respectively. In conclusion, Ubigo-X outperformed existing tools in MCC (for both balanced and unbalanced data) and AUC and ACC (for balanced data), highlighting the efficacy of integrating image-based feature representation and weighted voting in ubiquitination prediction. Ubigo-X is a potential species-neutral ubiquitination site prediction tool, accessible at http://merlin.nchu.edu.tw/ubigox/.

## Graphical Abstract

**Figure:**
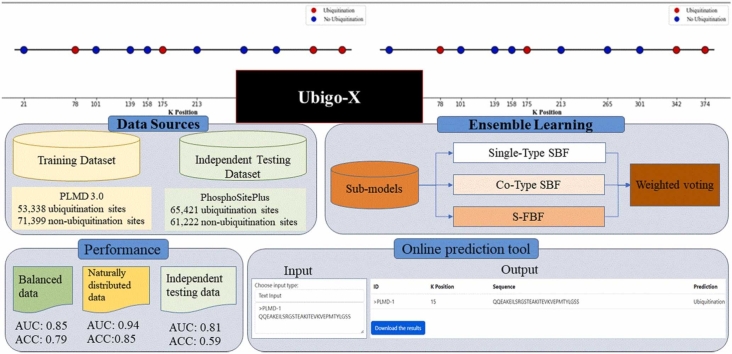

## 1 Introduction

Ubiquitin is a small, highly conserved 76-residue protein in eukaryotes [1]. Ubiquitin can exist as an unconjugated protein or be conjugated to other proteins, either as a single ubiquitin molecule or as part of a polyubiquitin chain [2]. Ubiquitination, characterized by the covalent attachment of ubiquitin to protein substrates, is a crucial post-translational modification that regulates various cellular functions [3], [4]. During this process, ubiquitin attaches to specific lysine residues on the protein substrates, resulting in transcriptional and translational changes. Ubiquitination involves three steps: activation, catalyzed by ubiquitin-activating enzyme (E1); conjugation, catalyzed by ubiquitin-conjugating enzyme (E2); and ligation, catalyzed by ubiquitin ligase (E3) [5], [6]. Identifying potential ubiquitination sites is crucial in understanding protein regulation and molecular mechanisms [7]. However, ubiquitination site identification using conventional biological experimental methods, such as mass spectrometry and antibody identification, is expensive and time-consuming [8], [9], [10]. Therefore, developing accurate and efficient tools for protein ubiquitination detection remains crucial.

Several researchers have studied protein ubiquitination sites via computational techniques such as machine learning models [11], [12], [13], [14], [15], [16]. For instance, in 2008, Tung and Ho developed a pioneering ubiquitination prediction system, UbiPred, using the support vector machine (SVM) algorithm and 31 selected physicochemical properties of amino acids [11]. In 2011, Chen and colleagues developed a protein ubiquitination site predicting tool, CKSAAP_UbSite, using the SVM and k-spaced amino acid pairs [13]. Furthermore, in 2013, Chen and team introduced hCKSAAP_UbSite, an improved model for predicting human protein ubiquitination sites. The tool uses a support vector machine classifier based on protein aggregation tendencies, comprising k-spaced encoding for amino acid pairs, dipeptides, amino acid identity, and aggregation propensity [12]. This progress highlights the continuous improvement of computational methods and their practical potential in effectively identifying protein ubiquitination sites. Integrating these models with different encoding schemes and utilizing advanced machine-learning techniques represents a significant step forward in bioinformatics and proteomics.

Deep learning has proven to be highly effective in analyzing complex structures in high-dimensional data in recent years. This effectiveness could be attributed to deep learning’s multi-layer networks and nonlinear mapping operations, which adapt well to complex data structures [17], [18]. Hongli and colleagues utilized four distinct features and developed DeepUbi, a convolutional neural network (CNN) deep learning method for predicting protein ubiquitination [19]. Liu and colleagues also proposed DeepTL-Ubi, a deep transfer learning-based predictor for multi-species ubiquitination sites [20]. Other researchers utilized different properties to build CNN models for ubiquitination site prediction, such as ‘HUbipPred’ [21], ‘Caps-Ubi’ [22], ‘ESA-Ubisite’ [23]. For instance, Luo and team developed ‘Caps-Ubi’ using convolutional and capsule networks and a hybrid of one-hot and amino acid encoding methods [22]. Wang and team developed ESA-Ubisite to predict ubiquitination sites in negative samples by applying SVM on physicochemical properties [23]. Table 1 summarizes some approaches and features previously used in building protein ubiquitination prediction tools.

**Table 1:** A summary of approaches and features used by previous protein ubiquitination prediction systems.

Prediction system	Approach used	Features used
UbiPred [11]	Support vector machine	Informative physicochemical properties of amino acids
CKSAAP_UbSite [13]	Support vector machine	Composition of k-spaced amino acid pairs surrounding any lysine in a query sequence
hCKSAAP_UbSite [12]	Support vector machine	K-spaced amino acid, binary amino acid, amino acid index physicochemical property, and protein aggregation propensity encoding
DeepUbi [19]	Convolutional neural network	One-hot encoding, informative physicochemical properties, composition of k-spaced amino acid pairs, and the pseudo amino acid composition
DeepTL-Ubi [20]	Densely connected convolutional neural network (DCCNN)	One-hot encoding of protein fragments
HUbipPred [21]	Ensemble method	Binary encoding and physicochemical properties of amino acids
Caps-Ubi [22]	Deep learning network	One-hot and amino acid continuous encoding
ESA-Ubisite [23]	SVM	Physicochemical properties of amino acids

Despite the progress in ubiquitination site prediction research, further work is needed to improve performance, especially when using naturally distributed data. Transforming biological sequences or structured data into image-like formats can uncover spatial and hierarchical relationships in the input data, enhancing CNN-based learning and classification performance [24], [25], [26], [27]. For instance, in one study, converting feature vectors into spatial image formats improved CNN-based drug response predictions [24]. In a genome classification study, CNNs trained on image-transformed sequences outperformed those trained on raw sequences [25]. In other studies, DNA sequences converted into image formats enabled CNNs to capture biologically meaningful spatial patterns [26], [27]. To our knowledge, the image transformation strategy has not been fully applied to protein ubiquitination prediction. In this study, we employ an image-based feature representation approach, where protein sequence features are transformed into image formats suitable for deep learning. Given the limited existing research on this concept and the critical role of images in deep learning, we propose Ubigo-X (http://merlin.nchu.edu.tw/ubigox/), a novel tool combining sequence-based, structure-based, and function-based features to enhance ubiquitination site prediction. Our ensemble strategy integrates deep learning based on image-transformed protein sequence features and traditional machine learning through weighted voting.

## 2 Materials and methods

### 2.1 Data collection

Sequence-based, structure-based, and function-based features were extracted from the training dataset, PLMD 3.0 [28]. The features included AAC [29], AAindex [30], one-hot encoding [31], basic k-mer [32], structural features such as secondary structure and solvent accessibility [33], and functional features such as signal peptide cleavage sites [34]. Missing sequences were replaced with the dummy amino acid ‘X.’ Initially, 25,103 protein sequences containing ubiquitination sites were extracted. To reduce redundancy, sequences with more than 30 % identity were removed using CD-HIT [35], resulting in a refined dataset of 12,753 protein sequences with 53,338 ubiquitination sites and 251,292 non-ubiquitination sites. The choice of the 30 % threshold aligns with recommendations by Chen and colleagues, who demonstrated its effectiveness in minimizing overfitting [36]. Yang and colleagues also employed this cutoff in their protein structure prediction study, underscoring its practical relevance across related applications [37]. Negative samples whose similarity with any positive sample was above 40 % were filtered out by CD-HIT-2d [35] to prevent interference between negative and positive samples. The final training set consisted of 53,338 positive and 71,399 negative sequences. The predictive performance of the constructed model was independently tested on PhosphoSitePlus data [38]. The analyzed set included 8662 protein sequences containing 65,421 ubiquitination and 61,222 non-ubiquitination sites retained after CD-HIT and CD-HIT-2d filtering (Supplementary Table 1).

### 2.2 Feature encoding

Extracted sequence fragments were converted into sequence-based, structure-based, and function-based features. The sequence-based features included amino acid composition (AAC) [29], amino acid property encoding (AAindex) [30], one-hot encoding [31], and basic k-mer [32]. The structure-based features included secondary structure and relative solvent accessibility/accessible surface area [33], while the functional features comprised signal peptide cleavage sites [34].

For one-hot encoding, categorical data were converted into numerical formats, with missing sequences replaced with the dummy amino acid ‘X.’ One-hot encoding is a commonly used technique in machine learning [31]. In protein research specifically, it transforms amino acid sequences into vectors that computational models can process. Special symbols are often included alongside the 20 standard amino acids in some cases, resulting in 21 categories. Each amino acid is represented as a 21-dimensional vector, with only one element set to 1 and the remaining amino acids set to 0. AAindex is a valuable resource that compiles numerical indices reflecting amino acids’ biochemical and physicochemical characteristics. This database plays a significant role in various bioinformatics applications, such as protein structure prediction, interaction analysis, sequence alignment, and machine learning [30]. It includes three categories: AAindex1 for single amino acids, AAindex2 for amino acid pairs, and AAindex3 for mutation matrices. Our study adopted the approach used in the UbiPred model [11], where each amino acid was encoded with 31 properties, resulting in a 31-dimensional feature vector.

Protein sequence representation employed the basic k-mer encoding method implemented in the Pse-in-One platform [39]. In the k-mer-based approach, sequences are parsed into overlapping fragments of length *k*, and the frequency of each unique k-mer is calculated to generate a feature vector that captures short-range dependencies between residues [40]. We used *k* = 2 [41], yielding a 400-dimensional vector for each sequence, corresponding to the 20 × 20 possible dipeptide combinations among the standard amino acids.

The relative solvent accessibility (RSA), absolute solvent-accessible area (ASA), and secondary structure of protein residues were determined using NetsurfP-3.0. NetsurfP-3.0 is an advanced tool that predicts various protein sequence characteristics, such as potential compositional disorder, solvent exposure, and secondary structure, providing valuable insights into the spatial organization of intracellular proteins [33]. The RSA measures the extent to which a residue is exposed to a solvent. The ASA was calculated using the predicted RSA by multiplying the RSA by the maximum ASA (maxASA) for the residue. For secondary structures, encoding was done using three output values, representing the likelihood scores for α-helix, β-strand, and random coil. Signal peptide prediction was done using SignalP 6.0 [42].

**Fig. 1:**
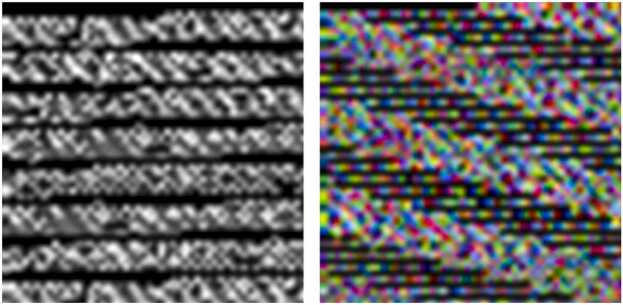
Grayscale and RGB images. Single-Type SBF features were transformed into image formats using two encoding schemes: grayscale (left) and RGB (right). Protein sequences were numerically encoded using AAC, one-hot encoding, and AAindex, and initially scaled to the 0–255 range for image construction. Grayscale images represented luminance values from a single feature dimension, while RGB images combined three feature sets across the red, green, and blue channels to enable multi-dimensional representation. Prior to model training, all image pixel values were normalized to a 0–1 scale to meet the input requirements of deep learning architectures such as ResNet34.

Based on the encoded features described above, three sub-models were developed to predict protein ubiquitination by leveraging different structural and functional properties (Table 2). The first sub-model (Single-Type SBF) focused on sequence-based features, including AAC, AAindex, and one-hot encoding. The second sub-model (Co-Type SBF) also used sequence-based features, specifically adopting k-mer representations generated using the Pse-in-One platform. Finally, the third sub-model (S-FBF) incorporated structure-based and function-based features due to their shared spatial and functional roles in regulating proteolytic processing and protein targeting [43], [44], [45]. For image-based representation, sequence-derived features (Single-Type SBF and Co-Type SBF) were arranged in matrices and converted into images to serve as inputs for deep learning. These images were initially normalized to the range [0, 255] for visualization in RGB and grayscale modes (Fig. 1). Before input into the deep learning model, all images were further scaled to the range [0, 1], following standard image preprocessing practices [46]. The RGB and grayscale images were compared as model inputs using the same hyperparameters.

**Table 2:** Overview of the three sub-models with their corresponding features and algorithms.

Sub model	Algorithm	Features
Single-Type SBF	ResNet34	Amino acid composition, AAindex encoding, and one-hot encoding
Co-Type SBF	ResNet34	K-mer alongside amino acid composition, AAindex encoding, and one-hot encoding
S-FBF	XGBoost	Secondary structure, relative solvent accessibility/absolute solvent-accessible area, and signal peptide cleavage site features.

**Fig. 2:**
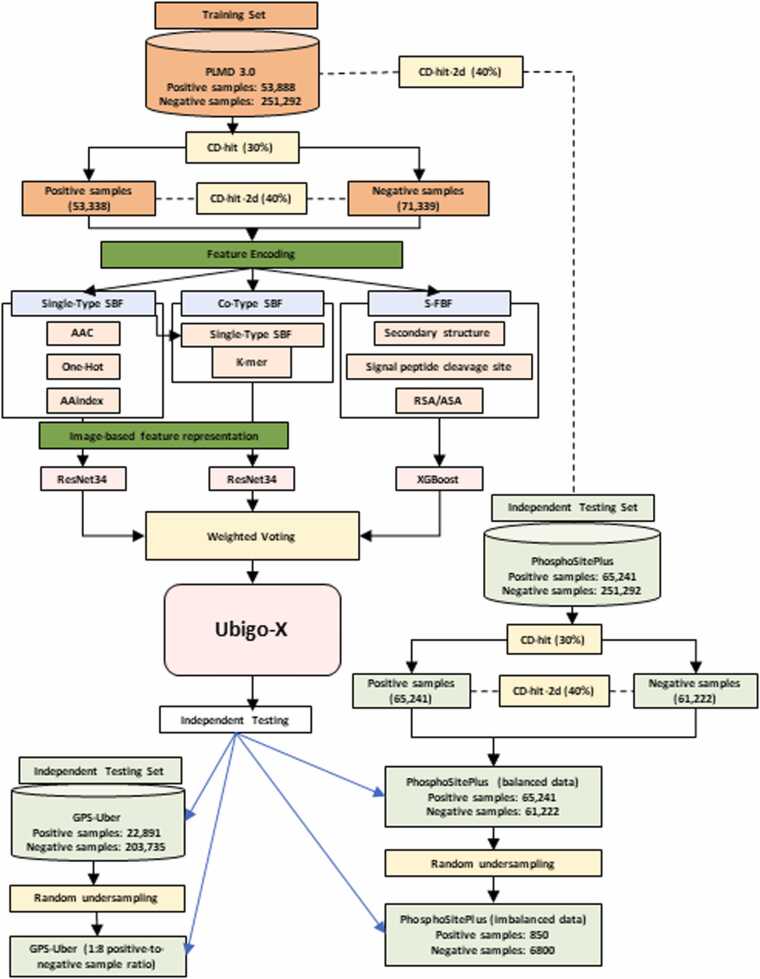
Study flowchart (Overview of the Ubigo-X workflow). The pipeline consists of four main stages: data preprocessing, feature extraction, model construction, and prediction. Training data were obtained from PLMD3.0 and filtered using CD-HIT (30 %) and CD-HIT-2d (40 %) to reduce redundancy and sequence similarity. Independent test sets from GPS-Uber and PhosphoSitePlus were similarly filtered to ensure non-overlapping evaluation. Three sub-models were developed, including Single-Type SBF (AAC, one-hot, AAindex), Co-Type SBF (k-mer), and S-FBF (structural and functional features). The Single-Type and Co-Type SBF models were trained using ResNet34, whereas the S-FBF model employed XGBoost for classification. Model outputs were integrated via a weighted voting ensemble. For evaluation, the original PhosphoSitePlus test set, which was balanced, was used alongside a separate, naturally distributed version to simulate real-world conditions. GPS-Uber was evaluated only in its original, imbalanced form.

### 2.3 Ensemble Learning and Strategies

**Fig. 3:**
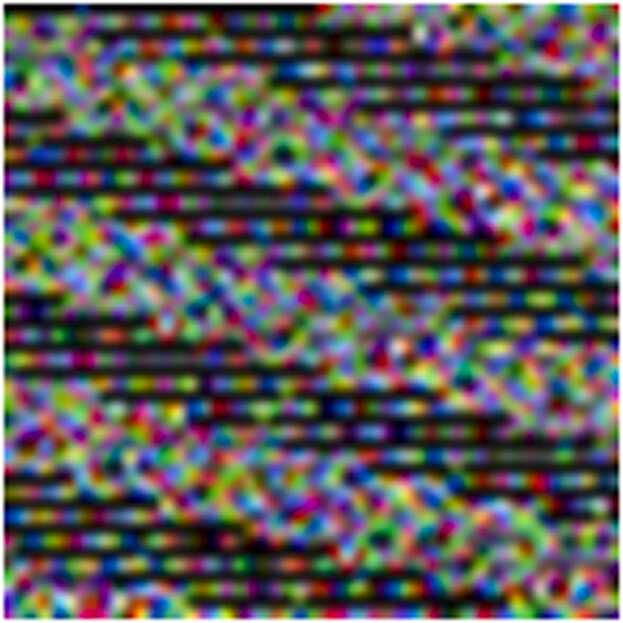
An image-based representation of Single-Type SBF. Single-Type SBF features (AAC, one-hot encoding, and AAindex) were combined and encoded across the red, green, and blue channels. This RGB format integrates all features into a unified multi-dimensional representation for prediction.

**Fig. 4:**
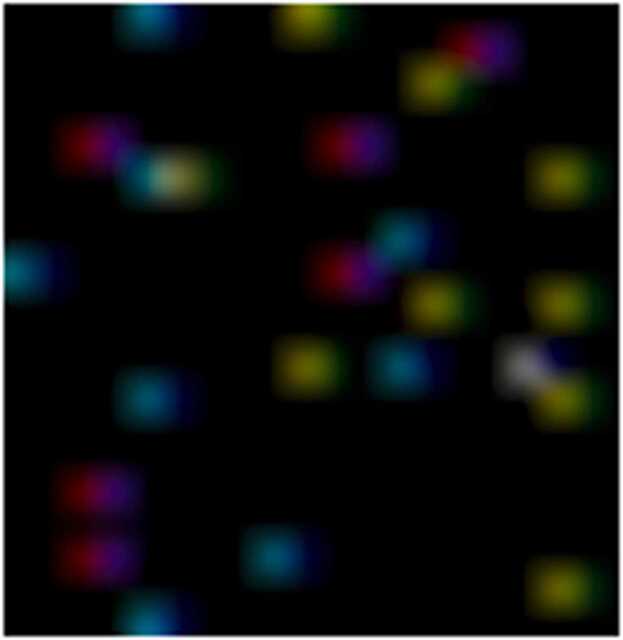
An image-based representation of Co-Type SBF. The figure shows RGB images generated from Co-Type SBF features based on k-mer representations of protein sequences. Each k-mer feature was converted and normalized to a 0–255 scale, then assigned to the red, green, or blue channel according to its weight. The colorful dots scattered throughout the images represent the weighted distribution and spatial arrangement of these k-mer features. This visual encoding captures multi-dimensional and positional information, allowing deep learning models to identify complex patterns and improve prediction accuracy.

Ensemble learning utilizes multiple models and their diversity and strengths. Even though it increases complexity and computational cost, it improves accuracy, robustness, and generalization capabilities with less overfitting than a single model [47]. The ensemble learning uses a weighted voting strategy, where multiple models (classifiers or regressors) make predictions based on assigned weights. In the current study, the three sub-models (Single-Type SBF, Co-Type SBF, and S-FBF), grouped based on characteristics that distinguished them, were first trained independently (separately). For the image-based models, including Single-Type SBF (Fig. 3) and Co-Type SBF (Fig. 4), the original training set was split into a new training set and a validation set in a 4:1 ratio. Each deep learning model was trained using a batch size of 8, a 0.001 learning rate, the Adam optimizer, and 30 epochs. Various deep learning algorithms, including ResNet34, DenseNet121, DenseNet201, Swin-base transformer, Swin tiny transformer, Vision-base transformer, and Vision large transformer, were initially tested to identify the most effective architecture for ubiquitination prediction. Based on the results, ResNet34 [48] was used in the final Single-Type SBF and Co-Type SBF models (Table 2). The XGBoost algorithm [49] was used in the S-FBF model (Table 2).

Ultimately, the ensemble (integrated) model comprised two ResNet34 algorithms for Single-Type SBF and Co-Type SBF and one XGBoost algorithm for S-FBF [48], [49]. ResNet34 used the binary cross-entropy (BCE) approach for loss value computation. BCE is well-suited for binary classification tasks [50]. The binary outputs (0 or 1) obtained from each sub-model were then integrated using a weighted voting ensemble approach [51]. The weights corresponded to each model’s accuracy on a 1:8 positive-to-negative dataset. A final classification was determined by summing the weighted outputs, with a threshold of 0.85 considered a positive prediction. This weighted voting scheme, utilizing the accuracies of the three sub-models (Single-Type SBF, Co-Type SBF, and S-FBF) as weights, was incorporated to develop Ubigo-X (Fig. 2).

### 2.4 Independent testing using naturally distributed data

The PhosphoSitePlus data used for testing comprised 65,421 ubiquitination sites and 61,222 non-ubiquitination sites, reflecting a nearly balanced dataset (Table 3). The nearly 1:1 distribution does not reflect the typical class imbalance naturally observed in ubiquitination data. To simulate a more realistic testing scenario, the original test set was downsampled using random undersampling without replacement from the positive class (ubiquitination sites), resulting in a 1:8 positive-to-negative distribution ratio [52]. This naturally imbalanced subset was used to evaluate the generalizability and robustness of Ubigo-X under practical biological conditions. The 1:8 approach aligns with the evaluation strategies used in the DeepUbi [19] and Caps-Ubi [22] models. The final imbalanced test set included 850 positive and 6800 negative ubiquitination sites, drawn from 99 protein sequences (Table 3). The number of sequences and site-level samples was determined based on the available positive examples, ensuring the desired class ratio and a representative evaluation size.

**Table 3:** Overview of the final training, validation, and testing data.

Set	Datasource	Number of ubiquitination (positive) sites	Number of non-ubiquitination (negative sites)
Training	PLMD3.0	53,338	71,339
Validation	PLMD3.0	10,668	14,267
Independent Testing (using balanced data)	PhosphoSitePlus	65,421	61,222
Independent Testing (using imbalanced or naturally distributed data)	PhosphoSitePlus	850	6800
Independent Testing (original data)	GPS-Uber	22,891	203,735
Independent Testing (downsampled data reflecting natural distribution)	GPS-Uber	1(ratio)	8(ratio)

### 2.5 Independent testing using a different database

Ubigo-X was evaluated using the GPS-Uber data [53] under two scenarios: the complete GPS-Uber dataset and the downsampled version. GPS-Uber is unique among ubiquitination site prediction tools because it was trained and tested on imbalanced datasets spanning multiple species. It provides an independent test dataset and an accessible online prediction tool [53]. First, Ubigo-X was tested on the complete, imbalanced GPS-Uber data, comprising 22,891 ubiquitination sites and 203,735 non-ubiquitination sites (Table 3). This large-scale evaluation assessed the generalizability of our model on biologically diverse external data. Second, a new independent test set consisting of 99 proteins was constructed by random undersampling without replacement to achieve a 1:8 positive-to-negative sample ratio (Table 3), reflecting a naturally imbalanced biological scenario. This smaller subset was submitted to both Ubigo-X and the GPS-Uber online tool for a more direct and rigorous comparison. Fig. 2 illustrates the overall workflow, while Table 3 shows the number of ubiquitination and non-ubiquitination sites in the training, validation, and testing sets.

### 2.6 Feature importance and model interpretability analysis

To address the interpretability of the models and gain insight into how different features influence predictions, we conducted a SHapley Additive exPlanations (SHAP) analysis on the independent test set using 1000 ubiquitination and 1000 non-ubiquitination sites. The SHAP model used the S-SBF(XGBoost), Single-Type SBF (ResNet34), and Co-Type SBF (ResNet34), allowing for a comparative analysis of feature impact across architectures. SHAP provides post-hoc explanations by assigning each input feature a SHAP value, quantifying its contribution to a specific prediction [54].

### 2.7 Model evaluation

Models were evaluated using six key metrics: accuracy, specificity (Sp), sensitivity (Sn), MCC, AUC, and loss value. Accuracy, specificity, sensitivity, and the MCC are usually derived from four fundamental components: true positive (TP), true negative (TN), false positive (FP), and false negative (FN). The mathematical expressions for these metrics are provided in the equations below:

$$\mathrm{ACC}=\frac{\mathrm{TP}+\mathrm{TN}}{\mathrm{TP}+\mathrm{TN}+\mathrm{FP}+\mathrm{FN}}$$

$$\mathrm{Sn}=\frac{\mathrm{TP}}{\mathrm{TP}+\mathrm{FN}}$$

$$\mathrm{Sp}=\frac{\mathrm{TP}}{\mathrm{TN}+\mathrm{FP}}$$

$$\mathrm{MCC}=\frac{\mathrm{TP}\times \mathrm{TN}-\mathrm{FP}\times \mathrm{FN}}{\sqrt{\left(\mathrm{TP}+\mathrm{FP})(\mathrm{TP}+\mathrm{FN})(\mathrm{TN}+\mathrm{FP})(\mathrm{TN}+\mathrm{FN}\right)}}$$

The AUC represents the value of a receiver operating characteristic (ROC) curve, quantifying the model’s ability to distinguish between classes. A higher AUC indicates better model performance. The loss value measures how well a model’s predictions match the actual target values, quantifying the difference between the predicted and actual outputs. The binary cross-entropy loss, which is well-suited for binary classification tasks, was used for ResNet-based analyses [50]. A lower binary cross-entropy loss indicates better model performance, while a higher value suggests less accurate predictions.

## 3 Results and discussion

### 3.1 Input methods for deep learning

Table 4 illustrates the performance of grayscale and RGB as input images under the same hyperparameters. The RGB images achieved better accuracy than grayscale images (0.694 vs. 0.606). The better performance of RGB could likely be due to several reasons. First, RGB images contain color information across three channels (red, green, and blue), while grayscale images only contain luminance information. Due to the increased dimensionality, RGB images provide more valuable information than grayscale images when using color information to distinguish between different categories or patterns [55]. Second, many deep learning models, such as ResNet and DenseNet, are often pre-trained on datasets containing RGB images, optimizing these models to process and interpret RGB input effectively [56], [57], [58], [59]. While these architectures can also handle grayscale images, their pre-trained weights are more suited for RGB data, contributing to the observed performance difference.

**Table 4:** Comparison of different input formats with the same epochs (training: validation = 4:1).

Input format	Binary cross-entropy loss	Validation accuracy
Grayscale	0.640	0.606
RGB	0.505	0.694

### 3.2 Comparison of algorithms and features for the single-type SBF model

Table 5 shows the performance of different algorithms in predicting protein ubiquitination using the Single-Type SBF model. The performances of the Swin transformer and Vision transformer models were below expectations. The accuracies were 0.572 for the Swin-base transformer and the Swin tiny transformer algorithms, 0.590 for Vision-base transformer and 0.562 for the Vision large transformer algorithm. The DenseNet models performed slightly better than the transformer models (ACC = 0.676 for Densenet121 and 0.683 for Densenet201). ResNet34 had the best performance (ACC = 0.688) and the least training loss (0.162), indicating better convergence [60]. Based on these results, ResNet34 was selected for use in subsequent models. ResNet34 outperformed other models probably because residual connections alleviate vanishing gradient problems and are suitable for handling large datasets, making the training of deep networks more stable [61].

**Table 5:** Performance of different classifiers in predicting protein ubiquitination using AAC, AAindex, and one-hot encoding for the Single-Type SBF Model.

Model (epochs = 30)	Loss (training)	Accuracy (validation)
DenseNet121	0.520	0.676
DenseNet201	0.581	0.683
ResNet34	0.162	0.688
Swin-base transformer	0.683	0.572
Swin tiny transformer	0.683	0.572
Vision-based transformer	0.670	0.590
Vision large transformer	0.694	0.562

Table 6 shows the performance of ResNet34 in predicting protein ubiquitination during independent testing using the Single-Type SBF model’s three features (AAC, one-hot encoding, and AAindex), independently and in combination. Individually, the features’ accuracies were 0.687, 0.690, and 0.686 for AAC, one-hot encoding, and AAindex, respectively. The respective sensitivities and specificities were 0.691 and 0.683 for ACC, 0.687 and 0.693 for one-hot encoding, and 0.659 and 0.714 for AAindex. Combining these features yielded the highest accuracy (0.700), with a sensitivity of 0.706 and specificity of 0.693. Due to this improved performance, the three features were ultimately combined as the Single-Type SBF.

**Table 6:** Independent testing performance of ResNet34 in predicting protein ubiquitination using AAC, one-hot encoding, and AAindex as features for the Single-Type SBF model.

Feature	Sensitivity	Specificity	Accuracy
AAC	0.691	0.683	0.687
One-hot encoding	0.687	0.693	0.690
AAindex	0.659	0.714	0.686
Combined	0.706	0.693	0.700

### 3.3 Comparison of features for co-type SBF

Table 7 summarizes the performance of various feature combinations using ResNet34 in Pse-in-One. K-mer showed the best performance across metrics for individual features, with a sensitivity of 0.684, specificity of 0.801, and accuracy of 0.740. For the combined features, k-mer and PC-PseAAC achieved a sensitivity of 0.607, a specificity of 0.770, and an accuracy of 0.686; k-mer and SC-PseAAC reached a sensitivity of 0.544, a specificity of 0.818, and an accuracy of 0.677; k-mer, PC-PseAAC, and SC-PseAAC yielded 0.552 sensitivity, 0.829 specificity, and 0.686 accuracy. Combining all features yielded 0.603, 0.792, and 0.694, corresponding to sensitivity, specificity, and accuracy. These results underscore the effectiveness of k-mer as an independent feature, particularly highlighting its superior performance in the ResNet34 model. Consequently, k-mer was the independent feature for Co-Type SBF.

**Table 7:** The performance of ResNet34 in Pse-in-One based on various feature combinations.

Feature	Sensitivity	Specificity	Accuracy
PC-PseAAC	0.342	0.857	0.592
SC-PseAAC	0.496	0.786	0.636
K-mer	0.684	0.801	0.740
Auto covariance	0.054	0.969	0.497
Cross covariance	0.163	0.898	0.519
Auto-cross covariance	0.186	0.890	0.527
K-mer + PC-PseAAC	0.607	0.770	0.686
K-mer + SC-PseAAC	0.544	0.818	0.677
K-mer + PC + SC-PseAAC	0.552	0.829	0.686
All features combined	0.603	0.792	0.694

### 3.4 Comparison of algorithms and features for the S-FBF model

Table 8 shows the performance of different models using a combination of signal peptides (SignalP-6.0 features) and secondary structure alongside RSA/ASA (NetsurfP-3.0 features) for the S-FBF model. DenseNet121 achieved the highest accuracy (0.601), followed by ResNet34 (0.597), Vision large transformer (0.583), Vision-base transformer (0.578), and Densenet201 (0.573). The Swin tiny transformer and Swin-base transformer models had the least accuracy (0.572). These observations underscore the significant impact of model architecture on prediction performance, as the combination of NetSurfP-3.0 and SignalP-6.0 features failed to yield consistent improvements across models. While feature enrichment remains crucial, the underlying model architecture is essential for improving prediction accuracy and overall performance.

**Table 8:** Performance of different models after combining NetsurfP-3.0 and SignalP-6.0 as features for the S-FBF model.

Model (epochs = 30)	Loss (training)	Accuracy (validation)
DenseNet121	0.661	0.601
DenseNet201	0.481	0.573
ResNet34	0.643	0.597
Swin tiny transformer	0.682	0.572
Swin-base transformer	0.683	0.572
Vision-base transformer	0.677	0.578
Vision large transformer	0.675	0.583

**Fig. 5:**
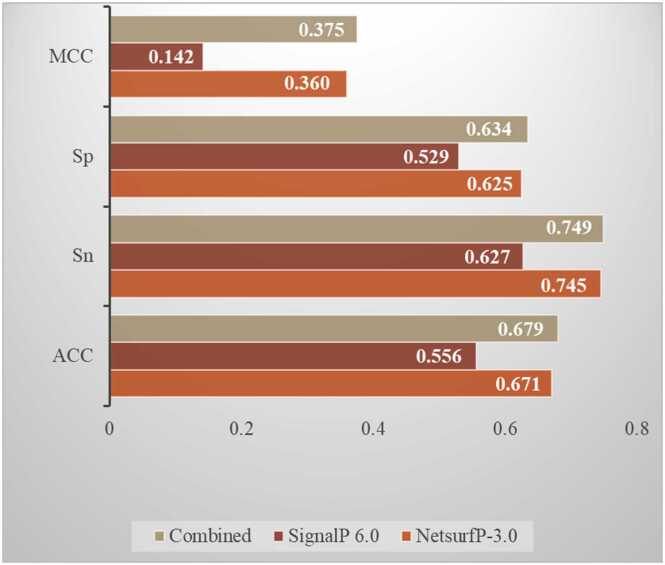
Performance of XGBoost in predicting protein ubiquitination using signal peptides and secondary structure+RSA/ASA as features. This figure compares the prediction performance of XGBoost using three different input configurations: SignalP 6.0 features (signal peptides), NetsurfP-3.0 features (secondary structure and RSA/ASA), and their combination. The performance metrics used were Matthews correlation coefficient (MCC), specificity (Sp), sensitivity (Sn), and overall accuracy (ACC). The combined feature set outperformed the individual sets across all metrics, achieving the highest MCC (0.375), specificity (0.634), sensitivity (0.749), and accuracy (0.679).

Fig. 5 presents the performance of XGBoost in predicting protein ubiquitination using SignalP 6.0 and NetsurfP-3.0 features individually and in combination for the S-FBF model. The combined feature category achieved superior performance across all evaluated metrics compared to the individual feature sets, with the highest MCC (0.375), indicating a strong correlation between predicted and actual classifications. The specificity was 0.634, showing a high true negative rate. The sensitivity reached 0.749, reflecting a high true positive rate. The accuracy was 0.679, highlighting the overall predictive capability. In contrast, the SignalP 6.0 feature set performed poorly (MCC = 0.142, specificity = 0.529, sensitivity = 0.627, and accuracy = 0.556). The NetsurfP-3.0 feature set performed slightly better than SignalP 6.0 (MCC = 0.360, specificity = 0.625, sensitivity = 0.745, and accuracy = 0.671). Therefore, combining SignalP 6.0 and NetsurfP-3.0 features has better predictive performance than using them individually. The improved performance likely stems from the complementary information from each feature set, enhancing the model’s ability to capture the underlying patterns in the data.

### 3.5 Independent testing

Table 9 presents the performance of Ubigo-X against other species-neutral ubiquitination site prediction tools using PhosphoSitePlus data. Ubigo-X outperformed several existing prediction models independently tested on balanced data. The accuracy, sensitivity, specificity, AUC, and MCC were 0.79, 0.74, 0.84, 0.85, and 0.58, respectively. While the sensitivity, specificity, ACC, and MCC for UbiPred [11] appear higher than those of Ubigo-X, both models have the same AUC. Notably, Ubipred solely used the traditional machine learning algorithm (i.e., the support vector machine), unlike Ubigo-X, which integrated both conventional machine learning and deep learning. Although Ubigo-X lagged behind tools such as DeepUbi [19] and Caps-Ubi [22] regarding individual metrics such as sensitivity, specificity, ACC, and AUC, its MCC was higher. Unlike other metrics that may be biased by class distribution, MCC provides a balanced evaluation of prediction performance across balanced and imbalanced data, indicating more reliable and consistent predictions [62], [63]. This improvement in MCC highlights the advantage of our ensemble approach, which integrates traditional machine learning and deep learning models to capture complex and discriminative feature representations better.

**Table 9:** Performance of Ubigo-X and other protein ubiquitination prediction tools during independent testing using balanced data from PhosphoSitePlus.

Method	Sensitivity	Specificity	Accuracy	MCC	AUC
CKSAAP_UbSite [13]	0.20	0.98	0.79	0.33	0.60
UbiNet [64]	0.72	0.60	0.64	0.31	-
UbiPred [11]	0.83	0.85	0.84	0.69	0.85
hCKSAAP_UbSite [12]	NS	NS	NS	NS	0.77
DeepTL-Ubi [20]	0.20–0.44	Fixed at 0.90 and 0.95	0.58–0.68	NS	0.72–0.89
HUbipPred [21]	0.81	0.73	0.77	0.55	0.84
ESA-Ubisite [23]	0.46	0.66	0.63	0.64	0.73
Multimodal deep architecture [65]	0.66	0.66	0.66	0.22	-
DeepUbi [19]	0.90	0.88	0.89	0.78	0.91
Caps-Ubi [22]	0.93	0.89	0.91	-	0.96
Single-Type SBF	0.71	0.69	0.70	0.40	0.70
Co-Type SBF	0.68	0.80	0.74	0.49	0.74
S-FBF	0.63	0.74	0.68	0.37	0.76
Ubigo-X	0.74	0.84	0.79	0.58	0.85

NS: not stated

### 3.6 Independent testing using naturally distributed (imbalanced) data derived from phosphositeplus

Table 10 shows our model performance during independent testing using an imbalanced data ratio (1:8). The three sub-models yielded higher sensitivities (0.79 for Single-Type SBF, 1.00 for Co-Type SBF, and 0.73 for S-FBF) than previous models, including DeepUbi (sensitivity = 0.46) and Caps-Ubi (sensitivity = 0.08). This exceptional performance demonstrates the effectiveness of our constructed models in predicting ubiquitination sites in proteins. Ubigo-X outperformed previous models across all key evaluation metrics, achieving a sensitivity of 0.91, accuracy of 0.85, AUC of 0.94, specificity of 0.84, and MCC of 0.55. The strong performance on naturally distributed (imbalanced) data reflects the model’s robustness in realistic biological settings, where positive samples are typically rare. The high sensitivity highlights Ubigo-X’s capability to detect true ubiquitination sites effectively. High sensitivity is especially valuable in large-scale screening or discovery-focused studies where minimizing false negatives is crucial. Nonetheless, elevated sensitivity may reflect a corresponding increase in the rate of false positives. Higher specificity may be required in clinical or resource-constrained experimental validation settings to reduce follow-up workload and cost. Ubigo-X addresses this need with a relatively high specificity of 0.84, indicating that it can correctly identify negative cases. This balance between sensitivity and specificity makes Ubigo-X adaptable for different application scenarios. Additionally, the model’s threshold can be adjusted to suit the specific requirements of a given task, whether prioritizing sensitivity for broad discovery or specificity for targeted validation.

**Table 10:** The comparison of different models using naturally distributed test data from PhosphoSitePlus.

Method	Sensitivity	Specificity	Accuracy	MCC	AUC
DeepUbi [19]	0.46	0.91	0.51	0.23	0.55
Caps-Ubi [22]	0.08	0.99	0.54	0.19	0.70
Single-Type SBF	0.79	0.48	0.51	0.16	0.63
Co-Type SBF	1.00	0.82	0.84	0.58	0.91
S-FBF	0.73	0.75	0.75	0.33	0.83
Ubigo-X	0.91	0.84	0.85	0.55	0.94

### 3.7 Testing using the GPS-uber database

Generally, all the sub-models (Single-Type SBF, Co-Type SBF, and S-FBF) performed well, with sensitivity significantly higher than specificity (Table 11). Since GPS-Uber only provides AUC, we could not thoroughly compare its ability to identify ubiquitination sites. GPS-Uber had a higher AUC than the sub-models. The AUC and sensitivity for Ubigo-X reached 0.81 and 0.90, respectively, demonstrating that Ubigo-X performs well across datasets with different ratios and sizes.

**Table 11:** Independent testing using the GPS-Uber dataset.

Model	Sensitivity	Specificity	Accuracy	MCC	AUC
GPS-Uber	-	-	-	-	0.76
Single-Type SBF	0.79	0.48	0.51	0.16	0.63
Co-Type SBF	0.86	0.60	0.63	0.28	0.73
S-FBF	0.77	0.54	0.56	0.18	0.70
Ubigo-X	0.90	0.55	0.59	0.27	0.81

Table 12 shows the prediction results for independent testing using the downsampled GPS-Uber. The three sub-models and Ubigo-X all outperformed GPS-Uber, implying that our model could be more effective than GPS-Uber in predicting protein ubiquitination based on imbalanced data ratios.

**Table 12:** Independent testing using our imbalanced data derived from GPS-Uber.

Model	Sensitivity	Specificity	Accuracy	MCC	AUC
GPS-Uber	0.45	0.84	0.80	0.24	0.65
Single-Type SBF	0.79	0.48	0.51	0.16	0.63
Co-Type SBF	1.00	0.82	0.84	0.58	0.91
S-FBF	0.73	0.75	0.75	0.33	0.83
Ubigo-X	0.91	0.84	0.85	0.55	0.94

The overall better performance of Ubigo-X in the imbalanced data setting (1:8 positive-to-negative ratio) for both PhosphoSitePlus and GPS-Uber can be attributed to Ubigo-X’s enhanced focus on the minority class, driven by the small number of positive samples. At the same time, the abundance of negative instances helps to sustain high overall accuracy. The relatively stable MCC value (0.55) further supports the model’s ability to generalize well in imbalanced settings, making it suitable for real-world biological prediction tasks. Unlike balanced datasets, where equal class representation may cause the model to overfit positive samples or underestimate the natural class distribution, the imbalanced setting better reflects real-world scenarios, allowing the model to learn more meaningful distinctions.

### 3.8 Model interpretability and feature contribution analysis

**Fig. 6:**
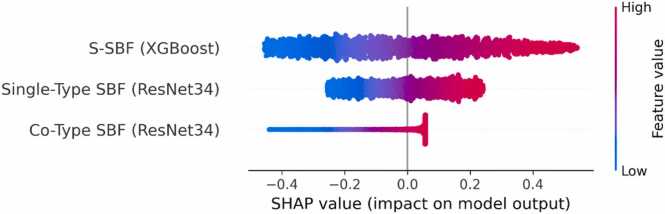
Interpretability analysis of feature contributions using SHAP values for XGBoost, ResNet (Single-Type SBF), and ResNet (Co-Type SBF). This SHAP (SHapley Additive exPlanations) summary plot visualizes the impact of input features on model output for three architectures. Each point represents an individual feature instance, with color indicating the feature value (blue = low, red = high) and position on the x-axis representing its SHAP value (impact on prediction). XGBoost and ResNet (Co-Type SBF) show broader SHAP distributions, reflecting diverse feature contributions across the input space. The ResNet (Single-Type SBF) displays a more compact SHAP spread, suggesting more consistent influence from its feature set. Together, these plots provide insight into how different feature encoding strategies influence model predictions and interpretability.

As shown in Fig. 6, we compared SHAP summary plots across S-FBF (XGBoost), Single-Type SBF (ResNet34), and Co-Type SBF (ResNet34). The Co-Type and S-BF models exhibited broader distributions of SHAP values, suggesting more diverse and distributed feature influence. In contrast, the Single-Type SBF model showed a more compact SHAP distribution, indicating consistent reliance on a subset of key features. This pattern reflects the differences in feature representation: while Single-Type SBF encodes one feature set at a time, Co-Type SBF integrates multiple complementary features, allowing the model to extract richer, multi-dimensional information.

Importantly, this analysis provides an interpretable view of the “black box” behavior often associated with deep learning models. It demonstrates that the Co-Type RGB representation improves predictive performance and enhances interpretability by revealing how high/low-value features impact predictions. Our incorporation of SHAP analysis offers researchers accurate predictions and mechanistic insight into model behavior, bridging the gap between performance and explainability in protein ubiquitination prediction.

## 4 Conclusion

In this study, we introduced Ubigo-X, an ensemble model that integrates machine learning and deep learning approaches for predicting protein ubiquitination sites. The model leveraged advanced features like AAC, one-hot encoding, AAindex for Single-Type SBF, k-mer for Co-Type SBF, and signal peptide with S-FBF. After incorporating weighted voting and image-based representation to enhance learning efficiency, Ubigo-X outperformed existing tools in predicting ubiquitination sites. Independent testing highlighted Ubigo-X’s robust generalization capabilities, with an accuracy of 0.85 and AUC of 0.94 on imbalanced data, outperforming benchmark models like GPS-Uber and Caps-Ubi. Although Ubigo-X incorporates deep learning components that require moderate computational resources during training (approximately 10 h on a standard GPU), the model is efficient during inference, enabling practical use even in modest computing environments. This balance between training cost and predictive performance supports its feasibility for broader adoption in the research community. Our findings emphasize the power of combining various feature representations and ensemble methods to improve prediction accuracy and robustness. The findings also highlight the ability of Ubigo-X to predict ubiquitination sites accurately across species. Hence, Ubigo-X is a potential tool for investigating ubiquitination across diverse biological contexts. Future research should focus on extending this approach to other post-translational modifications and enhancing model scalability for large datasets.

## Abbreviations

Ub: ubiquitin, PLMD: Protein Lysine Modification Database, SBF: sequence-based features, FBF: function-based features, AUC: area under the curve, ACC: accuracy, SVM: support vector machine, CNNs: convolutional neural networks, DCCNN: densely connected convolutional neural network, AAC: amino acid composition, AAindex: amino acid property encoding, RSA: relative solvent accessible area, ASA: absolute solvent accessible area, MCC: Matthews correlation coefficient, TP: true positive, TN: true negative, FP: false positive, FN: true negative, Sn: sensitivity, Sp: specificity, ROC: receiver operating characteristic

## CRediT authorship contribution statement

**Disline Manli Tantoh:** Writing – review & editing, Visualization, Validation, Methodology. **Ching-Hsuan Chien:** Visualization, Validation, Software, Methodology. **Jen-Chieh Yu:** Validation, Software, Methodology. **Yen-Wei Chu:** Writing – review & editing, Validation, Supervision, Software, Resources, Methodology, Funding acquisition. **Wei-Yi Yeh:** Writing – original draft, Visualization, Validation, Software, Methodology, Formal analysis, Conceptualization.

## Funding

This work was supported by funds from the National Science and Technology Council, Taiwan (grant numbers:111-2221-E-005-073-MY3, 113-2321-B-006-014, 112-2634-F-005-002, and 111-2423-H-006-002-MY3) and National Chung Hsing University-Changhua Christian Hospital project (grant number: NCHU-CCH 11307). The funder had no role in the study design, data collection, analysis, interpretation, manuscript writing, or the decision to submit for publication.

## Declaration of Competing Interest

The authors declare that they have no known competing financial interests or personal relationships that could have appeared to influence the work reported in this paper.

## Footnotes

Appendix A Supplementary data associated with this article can be found in the online version at doi:10.1016/j.csbj.2025.07.025.
