# Supplementary material for: Ubigo-X: Protein ubiquitination site prediction using ensemble learning with image-based feature representation and weighted voting
Source: Comput Struct Biotechnol J. 2025 Jul 14;27:3137–46. doi: 10.1016/j.csbj.2025.07.025 (PMC12303043; doi:10.1016/j.csbj.2025.07.025)
Supplement: Supplementary file 1 — Supplementary material [file mmc1.docx]

**Supplementary Table 1. The number of positive (ubiquitination sites) and negative samples (non-ubiquitination sites) in the training and testing sets before and after redundancy removal.**

| Stage | Training Set (PLMD v3.0) | | Testing Set (PhosphoSitePlus) | |
| --- | --- | --- | --- | --- |
|  | Number of protein sequences | Number of positive /negative samples | Number of protein sequences | Number of positive /negative samples |
| Before filtering | 25,103 |  | 19,863 |  |
| After redundancy removal using CD-HIT (>30% identity) | 12,753 | 53,338/251,292 | 8,662 | 65,421/251,292 |
| After filtering negative samples using CD-HIT-2d (> 40% similarity with positive samples) | 12,753 | 53,338/71,399 | 8,662 | 65,421/61,222 |
| Final analyses | 12,753 | 53,338/71,399 | 8,662 | 65,421/61,222 |
